# A retrospective analysis of oral and maxillofacial lesions in children and adolescents reported in two different services

**DOI:** 10.4317/jced.58231

**Published:** 2021-09-01

**Authors:** Anael-Sá-Costa-Borges de Almeida, Camila-de Nazaré-Alves-de Oliveira Kato, Humberto Jácome-Santos, João-de Jesus-Viana Pinheiro, Ricardo-Alves Mesquita, Lucas-Guimarães Abreu

**Affiliations:** 1 Graduate Student, Department of Oral Pathology and Oral Surgery, School of Dentistry, Universidade Federal de Minas Gerais, Belo Horizonte, Brazil; 2 Professor, Laboratory of Pathology and Immunohistochemistry (LAPI), School of Dentistry, Universidade Federal do Pará (UFPA), Belém-PA, Brazil; 3 Professor, Department of Oral Pathology and Oral Surgery, School of Dentistry, Universidade Federal de Minas Gerais, Belo Horizonte, Brazil; 4 Professor, Department of Child’s and Adolescent’s Oral Health, School of Dentistry, Universidade Federal de Minas Gerais, Belo Horizonte, Brazil

## Abstract

**Background:**

Most epidemiological studies involving oral and maxillofacial lesions assess only data from histopathological analysis. This may lead to a poor notification of diseases whose diagnosis is predominantly clinical. Aim: To evaluate and to compare the frequency of oral and maxillofacial lesions in children and adolescents in two different types of services: Oral Medicine clinic service and laboratory service.

**Material and Methods:**

The records of patients ≤ 19 years attending the Oral Medicine clinic service and records from the laboratory service in Oral and Maxillofacial Pathology of the Dental School of a university were analyzed.

**Results:**

828 records from the Oral Medicine clinic service and 2,409 records from the laboratory service were analyzed. The most common lesion group in both services was inflammatory/reactive lesions; however, infectious lesions and variations of normality were more frequently in the clinical service. Mucocele was the most common lesion in both services. The lips (28.9%) were the most affected region in the clinical service, while in the laboratory service, the bones (34.7%) were the most affected region.

**Conclusions:**

Some differences may occur with respect to the frequency of oral and maxillofacial lesions among pediatric individuals when data from different sources are compared.

** Key words:**Epidemiology, oral and maxillofacial pathology, oral medicine, oral and maxillofacial lesions, children, adolescents.

## Introduction

Oral health is essential for the overall health and quality of life of an individual. It is defined as “multifaceted and includes the ability to speak, smile, smell, taste, touch, chew, swallow, and convey a range of emotions through facial expressions with confidence and without pain, discomfort and disease of the craniofacial complex” (1). The stomatognathic system may be affected by several conditions (2,3), varying according to the age of the affected individual (2,4).

Children and adolescents are individuals with peculiar characteristics. They have different lifestyles and engage in different risky behaviors when compared to adults or older. Thus, they may be affected by specific diseases and illnesses (5). Knowledge about oral and maxillofacial lesions that affect these individuals is important for oral health practitioners as well as physicians in general practice and in Pediatrics (6), who will be able to provide an assertive diagnosis and timely referral to treatment, mitigating distress to the affected individual and his/her parent/caregiver (7). Moreover, awareness of the most frequent oral and maxillofacial lesions among young individuals may guide decisions during the development of oral health policies and allocation of resources in health facilities, where oral health services are provided to children and adolescents (8).

Epidemiological studies with representative samples about the frequency of oral and maxillofacial lesions in children and adolescents are yet to be fully explored in the literature. Usually, the outcomes evaluated in young individuals are dental caries, malocclusion, and traumatic tooth injuries (2,4,9,10). Moreover, previous studies through which the frequency of oral and maxillofacial disorders among pediatric individuals has been investigated are based only on the analysis of data from laboratory services (2,4,7,9,11,12). Data on oral and maxillofacial lesions that do not require biopsy and laboratory analysis for diagnosis have been poorly reported thus far. This may lead to the missing notification of diseases whose diagnosis is predominantly clinical.

Thus, considering the importance of analysis and knowledge about the frequency of oral and maxillofacial lesions in pediatric patients, the objective of this study was to evaluate and to compare the frequency of oral and maxillofacial lesions in children and adolescents in two different types of services (Oral Medicine clinic service and laboratory service). The findings of this original study were also compared to data extracted from a literature review.

## Material and Methods

• Step 1: Original study

-Study design and ethical issues

This retrospective study was approved by the Human Research Ethics Committee of the Federal University of Minas Gerais (CAAE: 10723019.0.1001.5149).

-Sample, eligibility criteria, setting and period of study

Records of individuals attending the Oral Medicine clinic service and records from the laboratory service in Oral and Maxillofacial Pathology of the Dental School of the Federal University of Minas Gerais, Belo Horizonte, Minas Gerais, Brazil, were retrieved and evaluated. The Oral Medicine clinic service is a referral center in Oral Medicine in the state of Minas Gerais, where services with respect to the diagnosis and treatment of oral and maxillofacial lesions to individuals from various regions of this state are provided. The laboratory provides services on histopathological and cytological analysis to the Oral Medicine clinic service of the university and to other public and private services in Brazil.

Records from 1990 to 2017 in the Oral Medicine clinic service and records from 1998 to 2018 in the laboratory service were retrieved. All records belonging to individuals aged ≤19 years were included. Individuals ≤9 years old were assigned to the group of children, and those ≥10 and ≤19 years old were assigned to the group of adolescents (13). Records with inconclusive diagnosis, duplicated records, cases of recurrence (same individual, same oral and maxillofacial lesion, same anatomical site, however, on different dates) and records with incomplete data regarding the age were excluded. Cases diagnosed in the laboratory service as hyperkeratosis with some degree of epithelial dysplasia were included in the lesion group of potentially malignant disorders. In the Oral Medicine clinic service, these lesions were classified as oral leukoplakia or actinic cheilitis (14).

-Diagnosis of oral and maxillofacial lesions

The diagnosis of oral and maxillofacial lesions was based on two criteria. Benign and malignant neoplasms were classified according to the 2017 World Health Organization (WHO) classification (15). The other conditions were classified according to the descriptions of the Oral and Maxillofacial Pathology textbook by Neville *et al*. (16). Then, the oral and maxillofacial lesions were assigned to ten different lesion groups, following a similar grouping method previously used by Fonseca *et al*. (17).

-Data collection

In addition to information on the diagnosis of the oral and maxillofacial lesion and the affected individuals’ age, information regarding the children’s/adolescents’ sex, skin color and anatomical location of the lesion was collected. Data related to the use of prosthesis in individuals affected by inflammatory fibrous hyperplasia, as well alcohol or tobacco use in children and adolescents affected by squamous cell carcinoma and hyperkeratosis with epithelial dysplasia were collected. Information related to the previous occurrence of trauma in individuals affected by mucocele and inflammatory fibrous hyperplasia was collected. Finally, data on any trauma to which the children or the adolescents had been submitted were also collected.

Data from the Oral Medicine clinic service, from the laboratory service and from the Oral Medicine clinic service and the laboratory service together were analyzed. For this last data pool, cases of individuals with records of the same oral and maxillofacial lesion (diagnosed on the same date) in both services, only the record of the Oral Medicine clinic service was computed. Records from the Oral Medicine clinic service are indicative of the final diagnosis of the oral and maxillofacial lesion after the accomplishment of a clinical exam and complementary exams, avoiding unreliable diagnoses in this dataset.

-Statistical analysis

Data were analyzed using the Social Package for Social Sciences (SPSS) program (SPSS, Inc., version 20.0, Armonk, NY, USA). Descriptive statistics and the chi-square test were performed. A comparison between the records of the Oral Medicine clinic service and the laboratory service regarding age, sex and skin color of children and adolescents was performed. Analyses of frequency for lesion groups in the Oral Medicine clinic service and in the laboratory service as well as among children and among adolescents were performed. Analyses of frequency of the ten most common oral and maxillofacial lesions in the Oral Medicine clinic service and in the laboratory service as well as among children and adolescents were performed. Analysis of the previous occurrence of trauma in individuals affected by mucocele and inflammatory fibrous hyperplasia in the Oral Medicine clinic service and in the laboratory service were performed. Analyses of frequency with respect to the anatomical locations that were most affected by oral and maxillofacial lesions in the clinical service, in the laboratory service, and in both services were also performed. Finally, analyses of frequency of the three most common oral and maxillofacial lesions in each of the lesion groups in the Oral Medicine clinic service, in the laboratory service and in both services as well as in children and adolescents were performed.

• Step 2: Literature review

-Source of information and eligibility criteria

A literature review aiming to identify retrospective studies assessing the frequency of oral and maxillofacial lesions in individuals aged ≤19 years was conducted in PubMed (National Library of Medicine). No publication language restriction was imposed. The terms and keywords used in the search are displayed in (Supplement 1) (http://www.medicinaoral.com/medoralfree01/aop/jced_58231_s01.pdf). The retrieved references were exported to EndNote software (Thompson Reuters, New York, NY, USA).

-Study selection

Study selection was performed in two stages. In Step 1, the titles/abstracts of the references were evaluated. References whose titles/abstracts fulfilled the eligibility criteria were included. For references whose titles/abstracts provided insufficient information for a decision on inclusion or otherwise, the full text was obtained and evaluated in Step 2. In this second step, after the full text assessment, references that met the eligibility criteria were also included.

-Data extraction and items extracted 

The following data were extracted from the articles included in the literature review: first author’s last name and year of publication, country where the study was conducted, sample size, origin of data (Oral Medicine clinic or laboratory service), age range (in years) of the affected individuals, the three most frequent oral and maxillofacial lesions evaluated, the most frequent group of oral and maxillofacial lesions evaluated and the most affected anatomical location.

## Results

• Step 1: Original study

The total number of records in both services was 32,842, of which 10,497 were from the Oral Medicine clinic service and 22,345 from the laboratory service. A total of 4,916 (14.9%) records belonged to individuals aged ≤19 years. Of these, 1,469 (29.8%) were from the Oral Medicine clinic service and 3,447 (70.2%) from the laboratory service. These values corresponded, respectively, to 13.99% of all Oral Medicine clinic service cases and 15.42% of all laboratory service cases. A total of 1,679 (34.1%) records were excluded after applying the eligibility criteria. Thus, 828 (25.5%) records of individuals who had attended the Oral Medicine clinic service and 2,409 (74.5%) records from the laboratory service were evaluated. When data of the Oral Medicine clinic service and the laboratory service were analyzed together, individuals with records in both services had data from the laboratory service excluded, resulting in a total of 3,040 records.

Table 1 shows the demographic data of the individuals who had attended the Oral Medicine clinic service and demographic data of individuals whose records were obtained from the laboratory service according to age, sex, and skin color. In the Oral Medicine clinic service (71.3%) and in the laboratory service (75.9%), most records belonged to adolescents (≥ 10 years). Female individuals were the most affected in the Oral Medicine clinic service (52.7%) and in the laboratory service (51.6%). There was a predominance of non-white individuals in the Oral Medicine clinic service (69.4%) and in the laboratory service (61.4%).

**Table 1:**
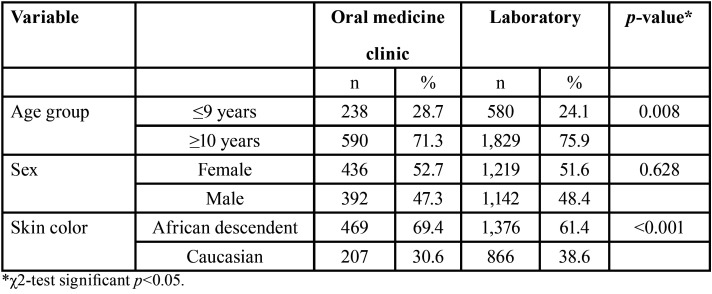
Demographic data observed in the records of the Oral Medicine clinic (n=828) and of the laboratory service (2,409).

The two most common lesion groups in the Oral Medicine clinic service and in the laboratory service were inflammatory/reactive lesions (1st) and odontogenic/non-odontogenic cysts (2nd). The third most frequent lesion group in the Oral Medicine clinic service was the group of infectious diseases. In the laboratory service, the group of benign neoplasms was the third most frequent. The group of variations of normality was the fifth most common in the Oral Medicine clinic service; however, this lesion group was uncommon in the laboratory service (9th) (Table 2).

**Table 2:**
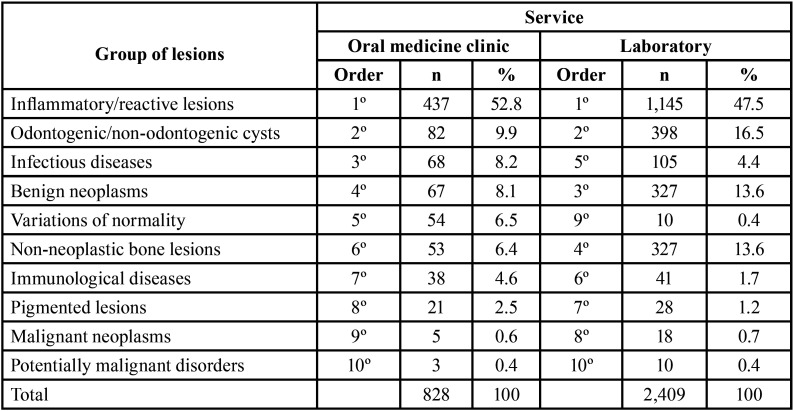
Frequency of the oral and maxillofacial groups of lesions in children/adolescents in the Oral Medicine clinic (n=828) and in the laboratory services (2,409)laboratory service (2,409).

Inflammatory/reactive lesions and odontogenic/non-odontogenic cysts were the two most common groups in children and in adolescents. Among children, 57.3% were affected by inflammatory/reactive lesions and 12.5% were affected by odontogenic/non-odontogenic cysts. Among adolescents, 44.0% were affected by inflammatory/reactive lesions and 16.4% were affected by odontogenic/non-odontogenic cysts. Benign neoplasms were the third most common lesion group in children (11.7%), while in adolescents, the group of non-neoplastic bone lesions was the third most common (15%) (Supplement 2) (http://www.medicinaoral.com/medoralfree01/aop/jced_58231_s02.pdf).

When comparing the frequency of lesion groups in children and adolescents in the Oral Medicine clinic service, some differences can be highlighted (Supplement 3) (http://www.medicinaoral.com/medoralfree01/aop/jced_58231_s03.pdf). Although the group of inflammatory and reactive lesions was the most common among individuals in both age ranges (53.8% in children and 52.4% in adolescents), infectious diseases was the third most common group among children (10.5%), and only the fifth among adolescents (7.3%). Moreover, in adolescents, benign neoplasms (8.6%, second most common group) and non-neoplastic bone lesions (8.1%, fourth most common group) were more frequent than in children (benign neoplasms: 6.7%, fourth most common group; non-neoplastic bones: 2.1%, seventh most common group). There were no cases of malignant neoplasms in children, while in adolescents, there were five cases.

Regarding the most common lesion groups in children and adolescents in the laboratory service (Supplement 4) (http://www.medicinaoral.com/medoralfree01/aop/jced_58231_s04.pdf)., although inflammatory/reactive lesions were the most common group among individuals in both age groups (60.7% in children and 43.4% in adolescents), some differences were found when individuals of the two age groups were compared. Different from the Oral Medicine clinic service (Supplement 3) (http://www.medicinaoral.com/medoralfree01/aop/jced_58231_s03.pdf)., in the laboratory service, benign neoplasms were more common in children (13.4%, second most frequent group) than in adolescents (13.6%, fourth most frequent group). In addition, non-neoplastic bone lesions were more frequent in adolescents (16.2%, third most frequent group) than in children (5.3%, fifth most frequent group).

The three most common lesions in the Oral Medicine clinic service were mucocele (25.9%), inflammatory fibrous hyperplasia (6.9%) and ranula (4.7%), while in the laboratory service, mucocele (25.6%), pericoronal follicle (9.5%) and dentigerous cyst (7%) were the three most common. Considering the analysis of data from both services together, the most frequent conditions were mucocele (24.5%), pericoronal follicle (7.5%) and inflammatory fibrous hyperplasia (6.6%) (Table 3).

**Table 3:**
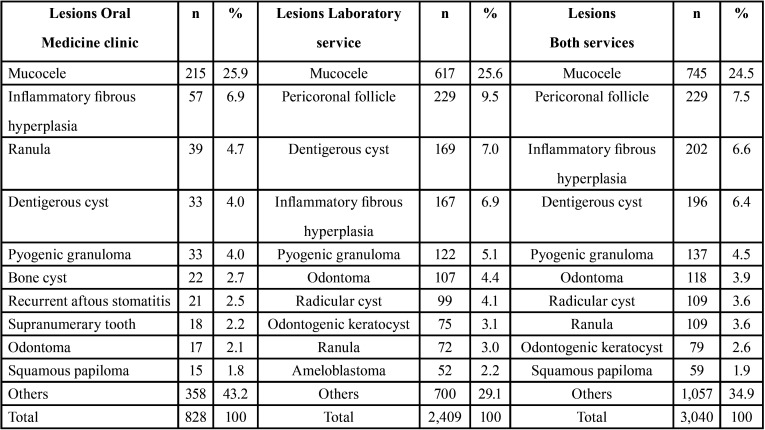
Frequency of the ten most common lesions of the Oral Medicine clinic (n=828), laboratory service (2,409) and both services (3,040).

Considering the analysis of both services together, the comparison between the ten most common lesions among individuals of each age group is showed in (Supplement 5) (http://www.medicinaoral.com/medoralfree01/aop/jced_58231_s05.pdf). Mucocele was the most frequent lesion among children (33.2%) and adolescents (21.6%). Among children, inflammatory fibrous hyperplasia (7.3%) and ranula (6.1%) were the other two most common lesions, while among adolescents, pericoronal follicle (9.5%) and dentigerous cyst (6.8%) were more frequent.

Of the 57 cases of inflammatory fibrous hyperplasia in the Oral Medicine clinic service, three (5.2%) individuals wore prosthesis. The records of the laboratory service did not present information about the wearing or the non-wearing of prostheses. Of the three cases of squamous cell carcinoma from the laboratory service, no individual was a smoker or used alcohol. Finally, of the nine cases of hyperkeratosis with atypia in the laboratory service, only one individual was a smoker and used alcohol. There were no cases of squamous cell carcinoma or leukoplakia in the Oral Medicine clinic service.

(Supplement 6) (http://www.medicinaoral.com/medoralfree01/aop/jced_58231_s06.pdf). displays information on trauma as an etiological factor of mucocele and inflammatory fibrous hyperplasia – the two most frequent inflammatory/reactive lesions in both services, either in a simultaneous analysis or in a separate analysis. Of the 215 cases of mucocele in the Oral Medicine clinic service, in 32 (14.88%) records there was a description of the occurrence of a previous traumatic episode. In the laboratory service, of the 617 cases of this same lesion, 16 (2.59%) records indicated the presence of this etiological factor. As regards inflammatory fibrous hyperplasia, of the 57 cases registered in the Oral Medicine clinic service, 16 (28.07%) records reported some kind of trauma. Of the 167 cases in the laboratory service, 16 (9.58%) records indicated some traumatic episode.

Table 4 shows the anatomical locations of oral and maxillofacial lesions. In the Oral Medicine clinic service, lips (28.9%), gingiva (11.2%) and bones (9.9%) were the most affected locations, while in the laboratory service, bones (34.7%), lips (28.1%) and tongue (7.6%) were the most affected locations. When data from both services were analyzed together, bones (29.3%), lips (27.3%) and tongue (7.7%) were the most affected anatomical locations by the lesions evaluated in this study.

**Table 4:**
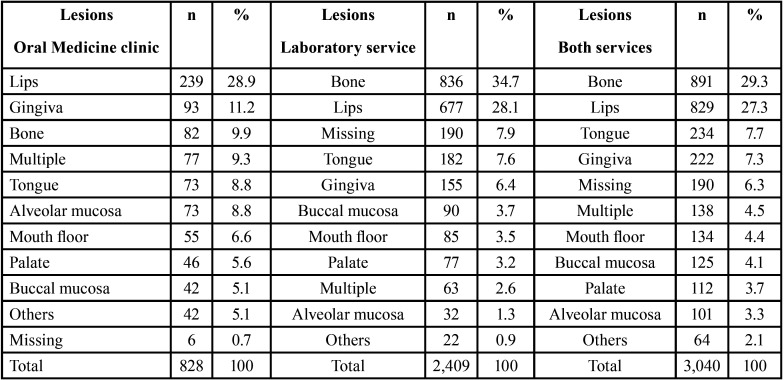
Location of the lesions of the Oral Medicine clinic (n=828), laboratory service (2,409) and both services (3,040).

The three most common oral and maxillofacial lesions in each of the lesion groups in the Oral Medicine clinic service, the laboratory service and in both services evaluated together are displayed in (Supplement 7) (http://www.medicinaoral.com/medoralfree01/aop/jced_58231_s07.pdf)., (Supplement 8) (http://www.medicinaoral.com/medoralfree01/aop/jced_58231_s08.pdf). and (Supplement 9) (http://www.medicinaoral.com/medoralfree01/aop/jced_58231_s09.pdf). The three most common oral and maxillofacial lesions in each of the lesion groups in children and in adolescents are showed in (Supplement 10) (http://www.medicinaoral.com/medoralfree01/aop/jced_58231_s010.pdf). and (Supplement 11) (http://www.medicinaoral.com/medoralfree01/aop/jced_58231_s011.pdf).

Step 2: Literature review

Search

We selected 11 articles (2,7,11,12,18-22,23,2),whose findings were compared to data of the original study.

-Characteristics of the studies included

The total population of the 11 included studies was 17,190 individuals. The studies were conducted in three continents: seven studies conducted in the Americas (2,7,11,12,20,23,24), three in Asia (18,19,22) and one in Europe (21). Six studies involving 9,691 individuals were conducted in Brazil.

-Results of the studies included

Table 5,  Table 5 cont. shows the characteristics and results of the studies included in the literature review. Mucocele was the most frequent lesion in seven included studies. Most frequent oral and maxillofacial lesion groups were reactive/inflammatory lesions (2,7,12,24), salivary gland pathology/disease (11,20,23) and cystic, odontogenic cysts, and cystic and tumoral odontogenic lesions (18,19,22). Regarding the most affected anatomical locations, the lips were the most affected: four included studies. Regarding the origin of the data, 10 studies had data retrieved in laboratory services and one from Oral Medicine clinic and laboratory services.

**Table 5:**
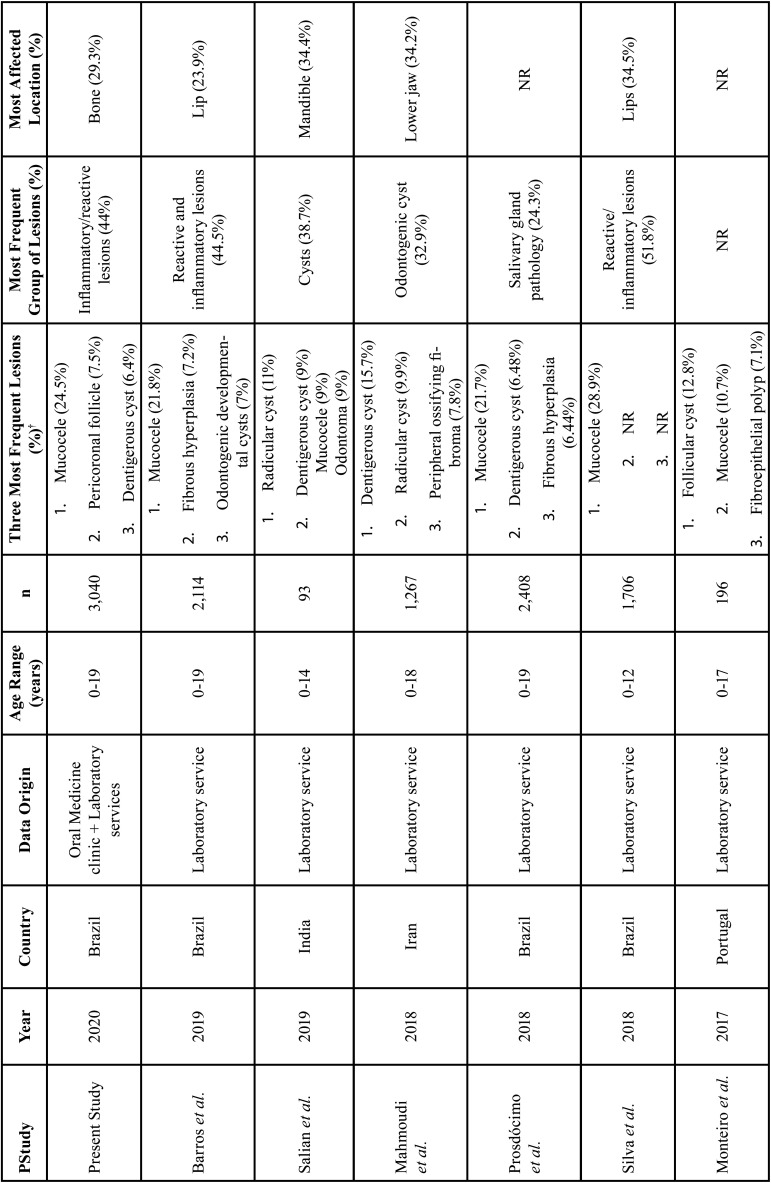
Characteristics and results of the original study and the studies included in the literature review.

**Table 5 cont.**
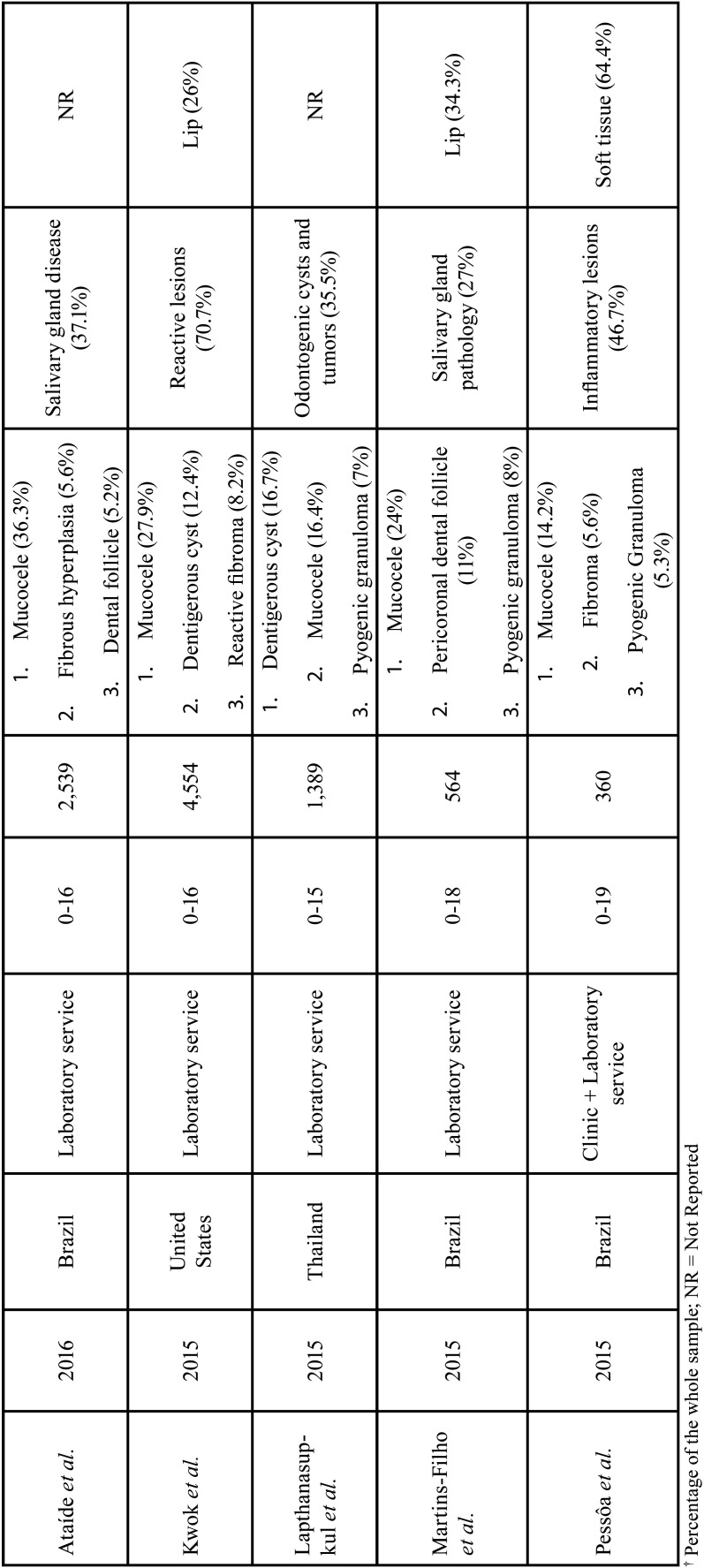
Characteristics and results of the original study and the studies included in the literature review.

## Discussion

There is a scarcity of studies on the frequency of oral and maxillofacial lesions in children and adolescents taking into account data from Oral Medicine clinic services and laboratory services. Studies are based exclusively on data from laboratory services. Only one study (24) analyzed data from laboratory and Oral Medicine clinic services, but no comparison between the two services was provided. Moreover, there is no uniformity among studies regarding the age groups in which children and adolescents were included (4,7,9,11,12). Therefore, restrictions regarding the data source and this diversity of age ranges adopted in the assessments precluded comparisons among studies.

Usually, samples of oral and maxillofacial lesions of pediatric populations correspond to 5.2% to 27% of all histopathological specimens of laboratory services of Oral and Maxillofacial Pathology (3,4,7,8,9,11,12). This variation may be a consequence of the heterogeneous distribution of diseases, sociodemographic characteristics of the populations in countries where the studies were conducted, genetic features of populations, different levels of exposure to etiological factors associated with the diseases, different characteristics of each service and different age groups or eligibility criteria for participation in the study (3,4). In our study, specimens of individuals aged ≤19 years accounted for 13.99% of all specimens of the Oral Medicine clinic service and 15.42% of all specimens of the laboratory service.

In our study, adolescents (≥10 years) were more affected by oral and maxillofacial lesions in comparison with children (≤9 years). Barros *et al*. (2), Ataide *et al*. (11) and Kwok *et al*. (12) also found that individuals during their adolescence were more affected by these lesions. This finding may be explained by the specific epidemiological characteristics of each disease and the strong preference of clinicians to postpone biopsies or surgeries in children, as they are aware that most oral and maxillofacial lesions involving these individuals are reactive or benign (11,24,25,26). Another possible explanation could be the greater exposure of this age group to mechanical trauma, as well as to etiological factors related to hygiene patterns, parafunctional habits and hormonal changes (8). It is noteworthy, however, that, in the present study, children were more affected by inflammatory/reactive lesions (57.3%) than adolescents (44.0%). In our study, female individuals were also slightly more affected by oral and maxillofacial lesions than male individuals, a finding also observed elsewhere (2,4,25). Finally, individuals of non-white skin color were more affected by oral and maxillofacial lesions, a result different from the findings of a Brazilian study (2), in which a higher frequency of oral and maxillofacial lesions among white individuals was observed. A possible explanation for this discrepancy may be the different sociodemographic characteristics of patients seeking oral health care in these two different dental facilities or also to the different methodology/concepts applied in these studies.

In our study, differences with respect to the comparison of data from the Oral Medicine clinic service with those from the laboratory service were observed. Regarding the most frequent lesion groups, infectious diseases was the third most common group (8.2%) in the Oral Medicine clinic service, while in the laboratory service, this group was the fifth in frequency (4.4%). This difference may have taken place because the diagnosis of some infectious diseases (for instance, labial herpes) is essentially clinical (16). Variations of normality was the fifth (6.5%) most frequent group in the Oral Medicine clinic service, while in the laboratory service, this group was the last but one (0.4%) in frequency. This difference may have occurred because biopsy procedure is, in most cases, unnecessary to diagnose the conditions of this group; clinical knowledge is often enough for this purpose.

Inflammatory/reactive lesions was the most frequent group of oral and maxillofacial lesions in the present study. It is important to highlight that there is no standardized classification of oral and maxillofacial lesions. However, our findings are in line with the results of most studies in the literature (2,4,7,12). In two Brazilian studies (11,25), the group of salivary gland diseases was the most frequent. In fact, this finding took place due to the occurrence of mucocele, the most frequent lesion in the evaluated studies. In these studies, mucocele was classified as a salivary gland outcome, while in our study and in other studies (2,7,9,12), mucocele was assigned to the group of inflammatory/reactive lesions. In a study conducted in Australia (26), the most common group of oral and maxillofacial lesions among pediatric individuals was pathology associated to the teeth. If this lesion group existed in our study, this would likely be one of the most frequent lesion groups, considering that the pericoronal follicle was the second most common lesion in our study (7.5%), when data of the Oral Medicine clinic service and of the laboratory service were analyzed together.

The second most frequent lesion group in the Oral Medicine clinic service and in the laboratory service was odontogenic/non-odontogenic cysts. Similar findings were reported elsewhere (2,7,26). Potentially malignant disorders and malignant neoplasms among children and adolescents were rare, corroborating the results of other authors (2,4,11,12). Three cases of actinic cheilitis were observed in the Oral Medicine clinic service, while in the laboratory service, ten cases of this group of oral and maxillofacial lesions were reported (nine cases of epithelial dysplasia and one of actinic cheilitis). Kwok *et al*. (12) stated that potentially malignant disorders and malignant lesions are significantly more frequent in adults than in children and adolescents. Etiological factors, such as nutritional deficiency, human papillomavirus (HPV) infection, sporadic mutations, and passive smoking may be associated with the occurrence of malignant neoplasms among the individuals of our sample (27,28).

In the Oral Medicine clinic service (25.9%) and in the laboratory service (25.6%), the most common oral and maxillofacial lesion was mucocele. This finding is in line with several other studies in the literature 11,12). In the study of Ataíde *et al*. (11), the frequency was 36.3%. The Figure in the study of Kwok *et al*. (12) was 27.91%. The etiology of this lesion is directly related to mechanical trauma, which is relatively common in pediatric patients (11). Although the scarce information on trauma detected in our study, this type of report was more common in the Oral Medicine clinic, suggesting that in this service, the amount of available information is more complete. Torabi-Parizi *et al*. (4) and Abdullah *et al*. (9) concluded that the most frequent lesion among children and adolescents was pyogenic granuloma. In our study, this lesion was the fifth most frequent among young individuals in the Oral Medicine clinic service and in the laboratory service.

Unlike other studies conducted in Brazil (3,25), in our study, ranula was more frequent in children (6.1%) than in adolescents (2.7%). Vale *et al*. (3) concluded that ranula affected more individuals in the second decade of life than in the first, while Cavalcante *et al*. (25) stated that the mean age of the individuals affected by ranula was 11.5 years. Recurrent aphthous stomatitis was the seventh most frequent oral and maxillofacial lesion in the Oral Medicine clinic service. However, such lesion was not among the most common lesions in the laboratory service. This difference occurs because, normally, the biopsy procedure to confirm the diagnosis is unnecessary.

The comparison of the ten most frequent oral and maxillofacial lesions in children and adolescents demonstrated that the pericoronal follicle was five times more frequent among adolescents than among children. This fact may have taken place due to extraction of third molars, a common procedure in late adolescence (29) and laboratory analysis of the specimen shortly after surgery for the removal of impacted teeth. Moreover, dentigerous cyst was also more common in adolescents (6.8%) than in children (5.4%). It is important to highlight that the development of these two conditions – pericoronal follicle and dentigerous cyst – is simultaneous to the development and eruption of permanent teeth, which contributes to the higher frequency of such conditions in adolescents. Ameloblastoma was also more frequent in adolescents than in children, which is in line with the literature (30). Finally, mucocele was more frequent in children (33.2%) than in adolescents (21.6%), which may be a possible explanation for the higher frequency of inflammatory/reactive lesions in individuals <10 years (57.3%) than in those who were between 10 and 19 years (44%).

Odontoma was the most common oral and maxillofacial lesion (31.63%) among the benign neoplasms, when data from the Oral Medicine clinic service and from the laboratory service were analyzed together. In the study carried out by Silva *et al*. (7), odontoma represented 26.6% of all benign neoplasms. Barros *et al*. (2) found a similar result in a study, in which 346 benign neoplasms were assessed. Of these, 77 (22.25%) were odontomas. Abdullah *et al*. (9) found that odontoma and ameloblastoma were the most common odontogenic tumors. Ataíde *et al*. (11), also stated that odontoma was the most frequent odontogenic tumor among the pediatric individuals evaluated in their study. It is noteworthy that odontoma is not acknowledged as a true neoplasm, even though this oral and maxillofacial lesion has been grouped as a benign neoplasia. According to the WHO, odontomas are tumor-like malformations of mixed epithelial and mesenchymal origin, composed of hard dental tissues and soft tissues (15).

Dentigerous cyst and radicular cyst (or periapical cyst) were the most common cystic lesions in the Oral Medicine clinic service (54.87% dentigerous cyst and 13.41% radicular cyst) and in the laboratory service (42.46% dentigerous cyst and 24.87% radicular cyst). In other studies, similar results were reported (7,11). It is also worth mentioning that in one Australian study (26), the dentigerous cyst was the most frequent oral and maxillofacial lesion in the entire sample. Some studies in Iraq and in the United Kingdom (9,10) have recognized the radicular cyst as the most common cyst in pediatric populations. This oral and maxillofacial lesion of inflammatory origin may indicate an impairment in oral health among children and adolescents, as they are associated with the progression of dental caries (2). Moreover, it is possible that the frequency of some periapical lesions, such as the radicular cyst is under-estimated because these lesions are not usually submitted to histopathological analysis (23).

Vascular malformations accounted for 22.95% of the variations of normality when data from the Oral Medicine clinic and the laboratory services were analyzed together. Although biopsy of this condition is contraindicated, the occurrence of this lesion was higher in the laboratory service, probably due to the nearly three times larger size of this dataset compared to the Oral Medicine clinic service dataset. The two most frequent lesions in the group of non-neoplastic bone lesions were pericoronal follicle (60.90%) and benign fibro-osseous lesions (12.23%). Silva *et al*. (7) stated that benign fibro-osseous lesions were the most frequent among non-neoplastic bone lesions, representing 31.5% of this specific lesion group. On the other hand, our results contrast with the findings of the studies of Abdullah *et al*. (9) and Ataíde *et al*. (11). In the first, juvenile ossifying fibroma was the most frequent non-neoplastic bone lesion. In the second, central giant cell lesion was reported as the most frequent. This difference may be due to the discrepancy in the classification of oral and maxillofacial lesions. For instance, in our study, pericoronal follicle was assigned to the group of non-neoplastic bone lesions, while in other studies 10,11), this lesion was assigned to the group of dental pathologies.

In the Oral Medicine clinic service, the lips were the anatomical location where most oral and maxillofacial lesions took place (28.9%). In the laboratory service, though, bones were the most affected anatomical location of oral and maxillofacial lesions (34.7%). This difference is related to the distinct nature of the services. Practitioners may send specimens of bone lesions for histopathological analysis. Osseous conditions as odontogenic keratocyst, ameloblastoma and benign fibrous osseous lesions demands histopathologic analysis for proper diagnosis (7). Moreover, some highly frequent lesions in the laboratory service, affecting bone regions, such as pericoronal follicle, radicular cyst, odontogenic keratocyst, and ameloblastoma were less observed in the Oral Medicine clinic service. Several other studies (2,3,7) also reported the lips as the anatomical location most affected by oral and maxillofacial lesions take placing in children and adolescents, portraying the high frequency of mucocele affecting the lower lips in young individuals (2,25). In a study carried out in Iran, the gingiva was the most affected location and mucocele was not among the three most frequent lesions (4). An American study (12) concluded that the most affected anatomical locations were the gingiva, lips, and mandibular areas. The latter was the second anatomical location most affected by oral and maxillofacial lesions in a Brazilian study (7). It is important to emphasize that there is no standardization regarding the analysis of anatomical locations affected by oral and maxillofacial lesions. In our study, for example, lesions affecting the mandible and the maxilla were assigned to the group of bone lesions.

## Conclusions

Our study has shown that some differences may occur during the assessment of data regarding the frequency of oral and maxillofacial lesions in children and adolescents from different sources (Oral Medicine clinic service and laboratory service), in particular with respect to infectious diseases and variations of normality. The frequency of these conditions could have been underestimated if only data from the laboratory service had been analyzed. We encourage further studies comparing the frequency of oral and maxillofacial lesions from Oral Medicine clinic service and from laboratory service in children and adolescents to confirm the results presented herein or otherwise. The nature of oral and maxillofacial lesions affecting adults is different from those typically found in children and adolescents, among whom asymptomatic inflammatory or reactive lesions are observed. Timely and assertive diagnosis and management of these lesions in pediatric populations is singularly important, as the craniofacial structures of these individuals will be submitted to growth and development. The presence of undiagnosed lesions that remain neglected or disorders with inadequate treatment may provoke disruptions during this period of consolidation of the craniofacial structures (6). Knowledge on the distribution of these lesions is of great importance, either for dentists, who have a privileged position in the early identification of these conditions or for physicians of allied specialties, who are frequently consulted by parents/caregivers.
